# Imaging fast electrical activity in the brain with electrical impedance tomography

**DOI:** 10.1016/j.neuroimage.2015.08.071

**Published:** 2016-01-01

**Authors:** Kirill Y. Aristovich, Brett C. Packham, Hwan Koo, Gustavo Sato dos Santos, Andy McEvoy, David S. Holder

**Affiliations:** a Department of Medical Physics and Bioengineering, University College London, Malet Place Engineering Building, Gower Street, London, WC1E 6BT, UK; b National Hospital for Neurology and Neurosurgery, University College London Hospitals, Queen Square, London, WC1N 3BG, UK

## Abstract

Imaging of neuronal depolarization in the brain is a major goal in neuroscience, but no technique currently exists that could image neural activity over milliseconds throughout the whole brain. Electrical impedance tomography (EIT) is an emerging medical imaging technique which can produce tomographic images of impedance changes with non-invasive surface electrodes. We report EIT imaging of impedance changes in rat somatosensory cerebral cortex with a resolution of 2 ms and < 200 μm during evoked potentials using epicortical arrays with 30 electrodes. Images were validated with local field potential recordings and current source-sink density analysis. Our results demonstrate that EIT can image neural activity in a volume 7 × 5 × 2 mm in somatosensory cerebral cortex with reduced invasiveness, greater resolution and imaging volume than other methods. Modeling indicates similar resolutions are feasible throughout the entire brain so this technique, uniquely, has the potential to image functional connectivity of cortical and subcortical structures.

## Highlights

• EIT can be used for imaging neural activity with < 200 μm and < 2 ms resolution.
• EIT features significantly correlate with CSDA.
• The spatiotemporal trajectory of activity can be traced using EIT.
• Imaging can be extended to the entire rat brain with a resolution of < 500 μm.

## Introduction

There is currently great interest in imaging depolarization and spiking in neuronal cell bodies or their processes in the brain, in order to validate and refine computational models of neuronal processing (Kopell et al., 2014, Sporns, 2014). A population level resolution of micrometers may be achieved with multiple microelectrodes as in the Utah array (Maynard et al., 1997) or optical methods such as two-photon imaging of calcium indicators or voltage sensitive optogenetic models (Ahrens et al., 2013, Hillman, 2007). Both optical methods have an imaging volume of < 1 mm^3^; microelectrode arrays sample over a few cubic millimeters (Kajikawa and Schroeder, 2011), but are penetrating and cause local tissue disruption. fMRI enables imaging throughout the brain, but images hemodynamic changes over seconds (Heeger and Ress, 2002), rather than true neural activity over milliseconds. Electrical impedance tomography (EIT) is a technique which has the unique potential to image fast neural activity over milliseconds throughout the brain, using non-penetrating surface electrodes.

In EIT, images are reconstructed from multiple measurements of transfer impedance made with surface electrodes. A single such measurement is usually made by injecting current at 1 Hz–1 MHz through a pair of electrodes and recording the resulting voltages at other electrodes. Several hundred measurements are made rapidly using electronic switching between electrodes (Bayford, 2006). Tomographic images of the internal electrical impedance are reconstructed, usually using a numerical geometric model of the subject, such as a finite element model (FEM), and inverse mathematical methods. The inverse solution is ill-posed and under-determined, and so regularization is required, without which small errors in measured voltages would result in large image artifacts. Images are reconstructed from the real component of the impedance and of differences over time, in order to minimize the effects of instrumentation and geometric errors (Lionheart, 2004). Unlike EEG inverse source modeling, EIT images have a unique inverse solution (Somersalo et al., 1992). EIT was originally developed for imaging the thorax (Henderson and Webster, 1978), and this was later extended to 3D tomographic imaging, with the aim of imaging lung ventilation (Metherall et al., 1996). It is now being used clinically to monitor lung ventilation (Frerichs, 2000, Luecke et al., 2012), and its use in imaging pulmonary perfusion (Nguyen et al., 2012), gastric emptying (Smallwood et al., 1994), breast (Zou and Guo, 2003) and prostate malignancy (Borsic et al., 2010) and acute cerebral stroke (Malone et al., 2014) is being investigated.

Impedance changes also occur as ion channels open during depolarization in neural tissue, which could potentially be imaged with EIT. These impedance changes were originally demonstrated in squid giant axons (Cole and Curtis, 1939), and subsequently confirmed during activity in cat cortex (Freygang and Landau, 1955, Klivington and Galambos, 1967), spinal motoneurons (Smith et al., 1967), and red nucleus neurons (Tsukahara and Fuller, 1969). Low-frequency current applied to neural tissue at rest predominantly travels in the extracellular space, and only penetrates cell membranes when ion channels open, resulting in an impedance decrease. With rising frequency, current increasingly crosses the capacitance of the cell membrane, which reduces the impedance change; impedance changes are about 1% when direct current (DC) is applied, and reduce to about 0.01% at 10 kHz (Liston et al., 2012, Oh et al., 2011). Until this study, it was not possible to produce accurate reconstructed images of fast neural activity, although advances in instrumentation and image reconstruction have now made such imaging possible (Aristovich et al., 2014, Oh et al., 2011).

Here we describe, for the first time, the implementation of EIT to image cortical neural activity, using a 30-electrode epicortical planar array. Impedance images of averaged evoked activity in the rat primary somatosensory cortex (S1) following mechanical stimulation of one of two whisker groups were produced. Images were validated with simultaneous local field potential (LFP) recordings and current source-sink density analysis (CSDA). Intrinsic signal optical imaging (ISOI) was employed to guide the placement of the EIT and LFP electrode arrays. Impedance images showed somatotopically separate activity for stimulation of the two whisker groups. Simultaneous electrophysiological recordings revealed correlation between EIT and CSDA, for activity onset time and depth (layer IV), and peak amplitude. Functional connectivity was extracted from impedance images using dynamic analysis. This revealed that the depth of largest lateral spread was at layer II/III, and occurred predominantly along barrel rows. This matched previously published findings (Lustig et al., 2013, Petersen et al., 2003). Our results demonstrate that EIT can image neural activity and major features of these match other established techniques, with reduced invasiveness, greater or comparable resolution and imaging volume (70 mm^3^) than other methods. While other techniques can match or surpass the individual features of EIT, none can match the unique combination of imaging on the mesoscopic scale, with high temporal resolution over large volumes. Simulations indicate that similar resolutions are feasible throughout the entire brain and so we anticipate EIT will enable mapping of functional connectivity of cortical and subcortical structures.

## Results

**Fig. 1:**
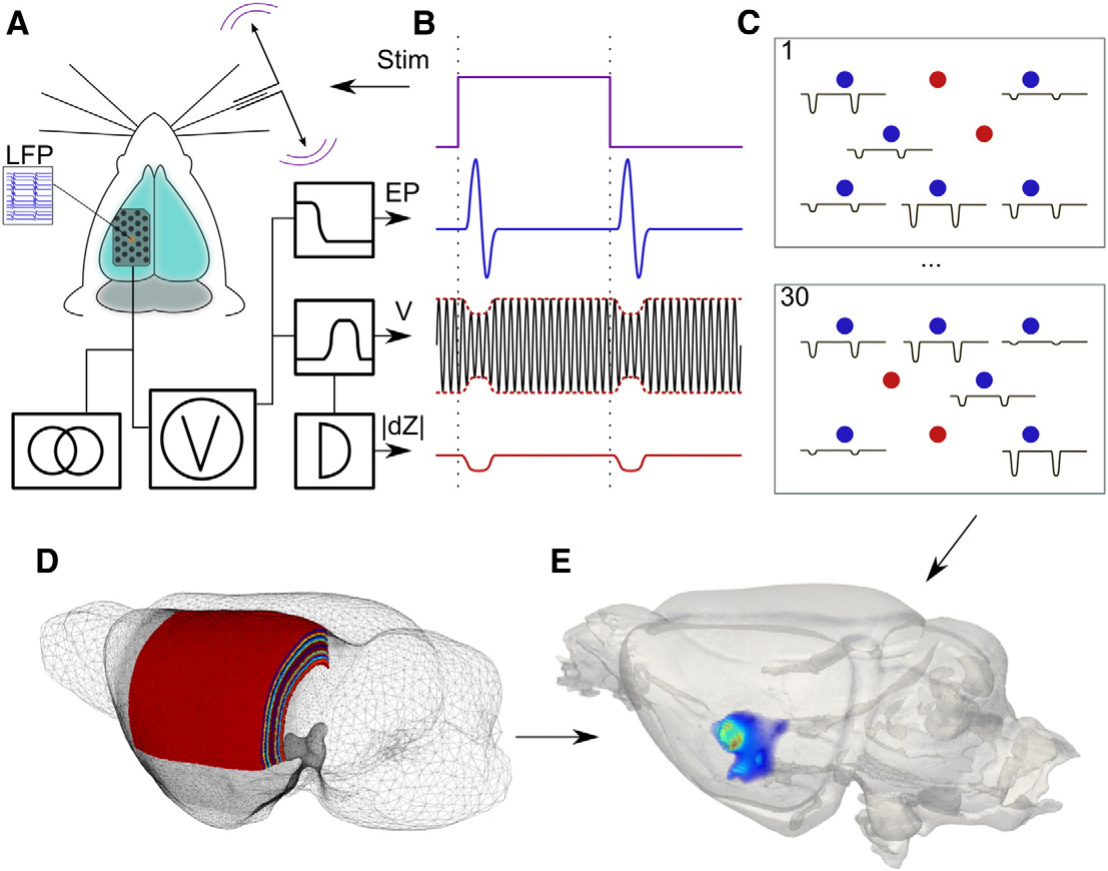
Method and paradigm. A) The 30 electrode array was placed over the exposed left S1 cerebral cortex. A 16 contact local field potential (LFP; orange dot) probe was placed through the center of the array over the activated whisker barrel group determined by intrinsic optical imaging. B) Impedance acquisition. The whiskers contralateral to the electrode array were moved forward and backward every second (stimulation waveform — Stim) and 15 cycles averaged for each impedance measurement. A constant amplitude current of 50 μA at 1725 Hz was injected through selected pairs of electrodes. The resulting voltages were recorded on all other 28 electrodes with respect to a reference in the contralateral scalp, low pass filtered at 400 Hz to yield evoked potentials (EP), and band pass filtered at 1725 ± 500 Hz to yield an amplitude modulated sine wave (V), which was demodulated to reveal the impedance change (|dZ|). C) This sequence was repeated for all 30 electrode injection pairs. The 1st and 30th injection pairs are illustrated. Red — injection pair; blue — resulting impedance decreases. Images were reconstructed using a 5 M element FEM tetrahedral mesh segmented into layers orthogonal to somatosensory cortex (D), and the resulting data stored in 4D spatiotemporal format (E).

In this work, EIT was undertaken using epicortical electrode arrays, placed over S1 in the anesthetized rat during evoked activity. The activity was induced using a piezoelectric stimulator, by 1 Hz mechanical displacement of diagonally adjacent groups of whiskers tied together: 1) δ, γ, E1, and D1, or 2) D2, C2, D3 and C3 (Diamond et al., 2008). Both groups were stimulated separately, twice in 4 rats, which yielded 16 image sets. EIT arrays comprised 30 platinized, stainless-steel electrodes embedded in silicone, with contacts 0.6 mm in diameter and centers offset in a triangular pattern 1.2 mm apart. The array was placed on the left cerebral hemisphere and centered upon the posteriomedial barrel subfield (PMBSF); the location of the PMBSF was determined prior to electrode placement using intrinsic signal optical imaging (ISOI). A 16-contact single shank LFP electrode array was placed in the center of the EIT electrode array. For EIT recordings, a sine-wave current (1.7 kHz and 50 μA) was injected through a single electrode pair at a time and the resultant voltages were recorded (Fig. 1A). The signal was filtered and demodulated with a bandwidth of ± 500 Hz, which gave a temporal resolution of 2 ms, to yield the evoked potentials (EPs) and the impedance change (δZ; Fig. 1B). The current injection pair was switched, using a multiplexer, every 15 s. This was repeated over 30 different electrode pairs in an expanding spiral pattern around the center of the array (Fig. 1C). Each complete image data set took c. 15 min. Data within each 15 s trial were averaged. The resulting c. 900 voltages were processed and used to produce images (Fig. 1D–E). LFP and EIT data were recorded simultaneously.

### EIT images and statistical analysis

**Fig. 2:**
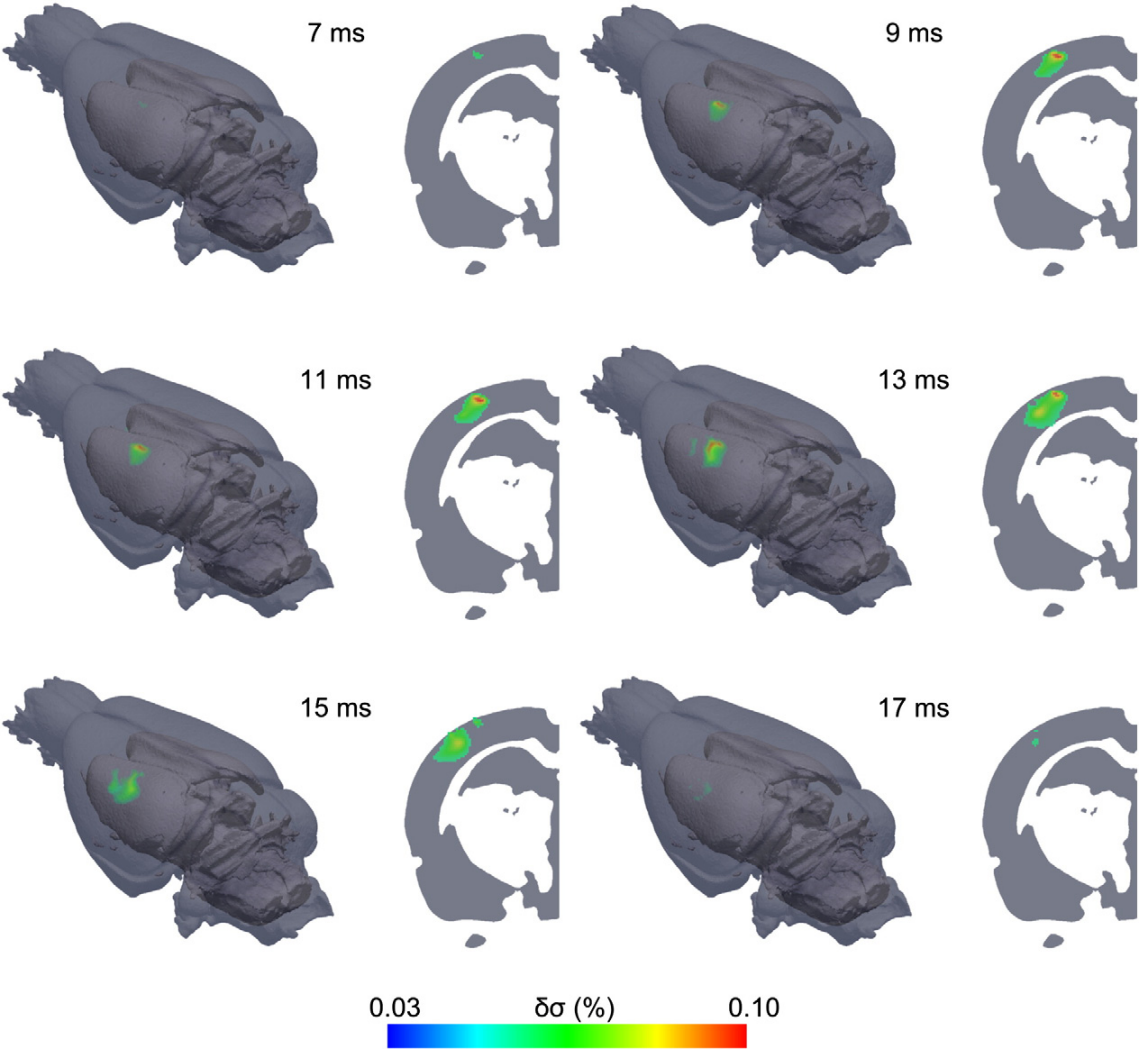
Example of EIT image during forward deflection of whisker group 1 (δ, γ, E1, and D1). The sequence of images (conductivity change — δσ), 2 ms apart, shows the onset of activity at 7 ms occurring at c. 800 μm beneath the pial surface, and over the ensuing milliseconds encompasses a larger volume reaching a maximum at 10–11 ms, following which the activity spreads to adjacent areas in S1 and disappears at 17 ms.

**Fig. 3:**
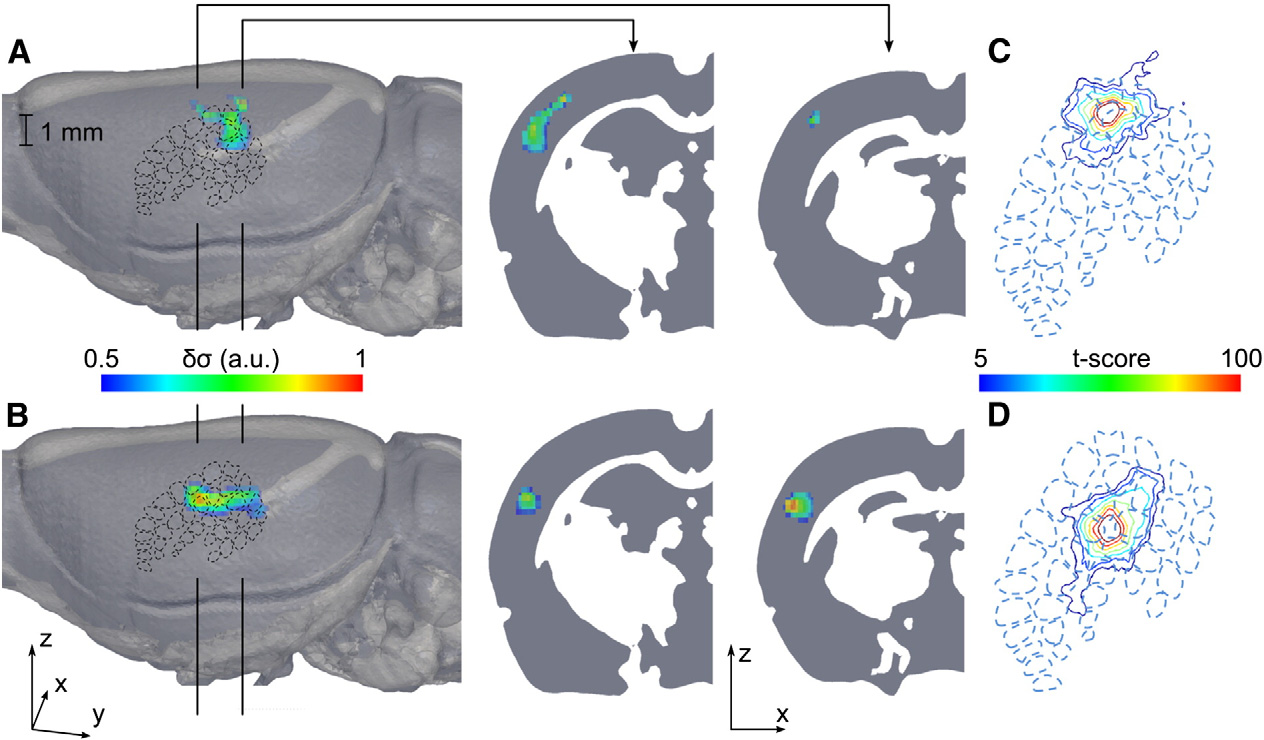
Population statistics of EIT images. A) and B) Grand average normalized conductivity change (δσ) at 8 ms for stimulation of whisker group 1 (δ, γ, E1, and D1), and group 2 (D2, C2, D3 and C3) respectively (*n* = 16 in 4 rats). C) and D) Significance map of peak δσ across rats and trials, projected onto the pial surface, across all layers (*n* = 16 in 4 rats) for group 1 and group 2 respectively.

Each data set was reconstructed into EIT images; reconstructions spanned 0 to 30 ms, with a 1 ms time step, where 0 ms was the time of stimulation. EIT images were visualized as percentage change in the conductivity with respect to the baseline (δσ). These images revealed that δσ arose in the neocortex approximately 800 μm beneath the array, and over the ensuing milliseconds encompassed a larger volume and reached maximum amplitude, following which activity then spread to adjacent areas in S1 (Fig. 2; Supplementary Video 1). Qualitative visual inspection of each image set (Fig. 2), grand average images of the normalized δσ for the two stimulation groups over repeats and subjects (*n* = 32, *N* = 4, Fig. 3A–B), and maps of the statistical significance of changes, expressed as a *t*-score (Fig. 3C–D) indicate that changes were confined to the PMBSF, although a larger region of somatosensory cortex was imaged. Reproducibility across recordings was assessed statistically by labeling active voxels using a binomial mask to identify the presence of activity. This indicated two spatially distinct areas of significance (*p* < 0.0001), one for stimulation of each whisker group. Both foci could be discriminated from one another by visual inspection (Fig. 3). This spatial confinement to stimulated barrels and differing somatotopic response for the two whisker groups suggests that the reconstructed changes were not artifactual. This was supported by control recordings (see Controls).

### Cross-validation with LFP recordings and CSDA

**Fig. 4:**
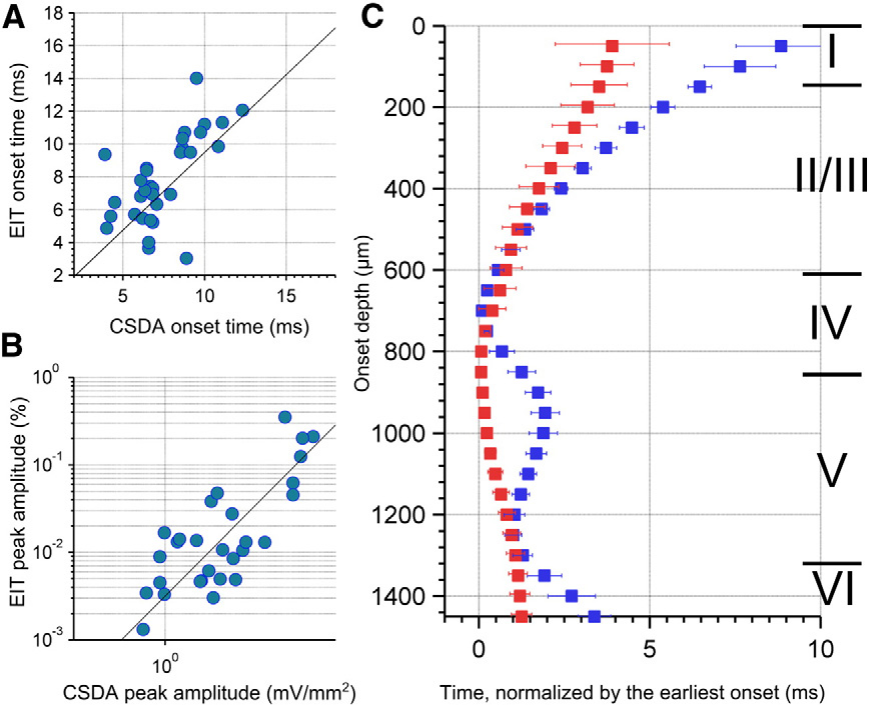
Cross-validation of EIT with CSDA. A) Correlation plot between EIT and CSDA onset time (*n* = 32, *N* = 4, *r* = 0.6, *p* < 0.001), B) correlation plot between EIT and CSDA amplitude (*n* = 32, *N* = 4, *r* = 0.95, *p* < 0.001), and C) the translaminar onset latencies for EIT (red) and CSDA (blue) over the depth, normalized by the time of earliest activity, mean (squares), and standard error (error bar).

The validity of imaged δσ was assessed by comparison with LFP changes recorded with a single shank 16 contact probe placed in the center of the EIT electrode array (Supplementary Fig. 2). The onset time of activity, the depth of the activity onset, and the amplitude of changes were extracted from EIT images and CSDA in a region 0.2 × 0.2 × 2 mm within which the LFP electrode had been placed (Table 1). The onset times for the activity in the EIT images and CSDA were significantly correlated (*n* = 32, *N* = 4, *r* = 0.6, *p* < 0.001, Fig. 4A). The onset times for EIT images were 0.6 ± 0.26 ms (mean ± SD, *n* = 32, *N* = 4) earlier than the onset time of activity within the CSDA. The onset of activity was located at a similar depth from the cortical surface for both EIT and CSDA, being 790 ± 70 μm and 730 ± 50 μm, respectively, which approximates layer IV and matches previous findings (Armstrong-James et al., 1991, Armstrong-James et al., 1992, Di et al., 1990, Einevoll et al., 2007, Petersen and Sakmann, 2001, Roy et al., 2011).The amplitudes of the conductivity changes in the EIT images and CSDA were also significantly correlated (Fig. 4B, *n* = 32, *N* = 4, *r* = 0.95, *p* < 0.001).

The translaminar onset latencies for both EIT and CSDA were assessed by comparing activity onset over depth against the depth at which activity was first detected, in 50 μm steps (Fig. 4C). The translaminar onset latencies for EIT were extracted from a region of interest (ROI) around the insertion site of the LFP electrode to allow for a more direct comparison of the findings of EIT and CSDA. At the location of the LFP electrode, δσ first appeared in layer IV (790 ± 70 μm), and then spread into layer VI (below 1400 μm) over 1.5 ms and superficially towards layer I (above 150 μm) over 3.5 ms. EIT activity onset was only significantly different (*p* > 0.05; paired *t*-test) from CSDA at the border of layers IV/V (850–1100 μm) and layer VI (below 1400 μm), CSDA activity occurred later by < 1.7 ms at these depths. δσ onset occurred progressively later with depth, while in the CSDA there were two separable fronts of activity onset. The second occurred at layer Vb, approximately 1200 μm, which was earlier than the activity above and below this site. These findings demonstrate clear similarities between activity measured with EIT and CSDA, with the exception of a clear delineation of activity onset in layer Vb.

### 4D trajectories of activity measured with EIT

**Fig. 5:**
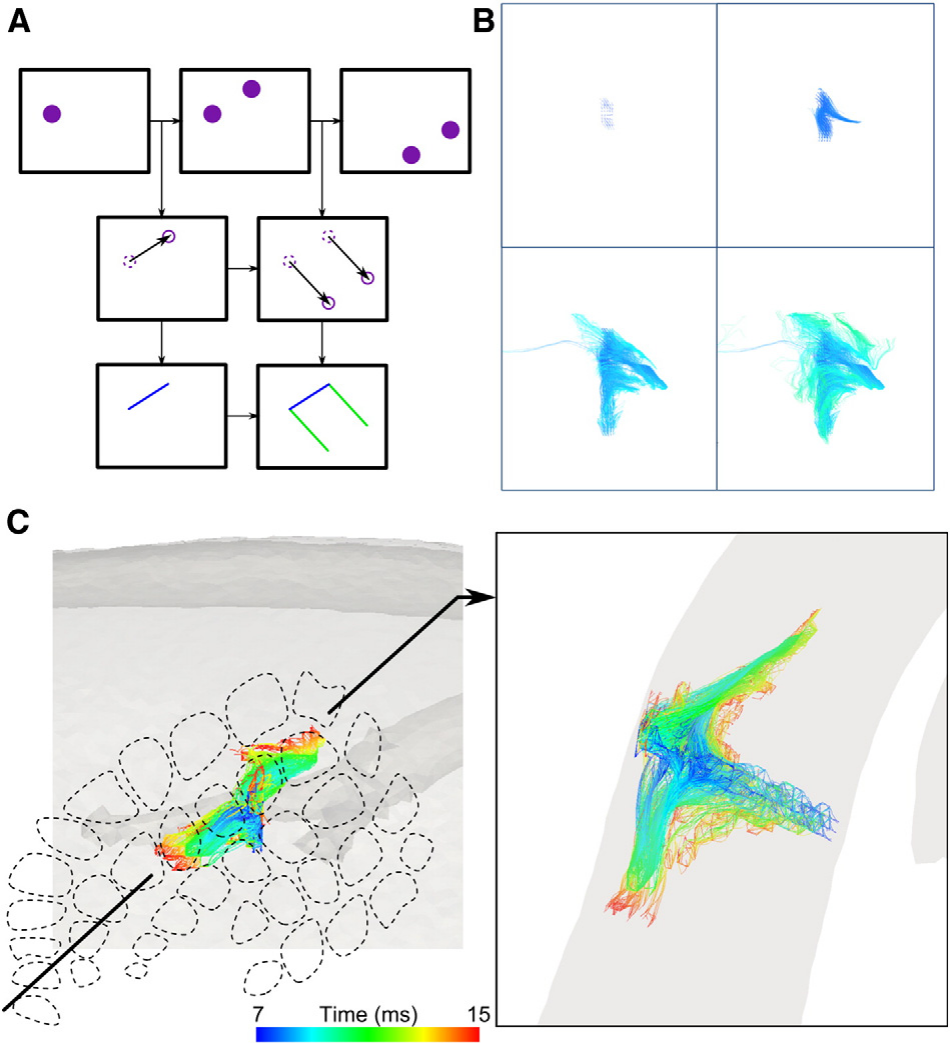
Example of spatiotemporal trajectories during forward deflection of whisker group 2 (D2, C2, D3 and C3). A) The trajectories have been computed with time sequenced images, using a particle filter approach, resulting in a B) set of 3D curves of activity propagation, color-coded with timing of activation over milliseconds. C) The surface (left) and coronal (right) views reveal that activity starts in layer IV, propagates into supra- and infragranular layers within the stimulated barrels, and then spreads to adjacent whisker barrel rows predominantly through layers II/III.

The 4D spatiotemporal propagation of activity was examined by computing 4D trajectories. Trajectories were reconstructed for each forward deflection image, as this was the preferred direction and yielded the largest amplitude results (Wilent and Contreras, 2005). These 4D spatiotemporal trajectories showed an early focus of activity around the stimulated whisker group. This core of activity occurred in the first 5 to 10 ms post-stimulus (Fig. 5). Where lateral spread of activity occurred, it was within the first 5 ms and any such lateral spread beyond the immediate surround whisker barrels was predominantly row-wise and in layers II/III (Fig. 5E; Supplementary Video 2). This also corresponds well with published findings (Lustig et al., 2013, Petersen, 2007, Petersen and Sakmann, 2001, Petersen et al., 2003). The depth at which the most connections occurred corresponded to layers II/III (450 ± 40 μm). This matches structural and functional findings of high levels of vertical and horizontal connectivity from layers II/III (Feldmeyer et al., 2006, Holmgren et al., 2003).

### Imaging neural activity throughout the brain in simulations

**Fig. 6:**
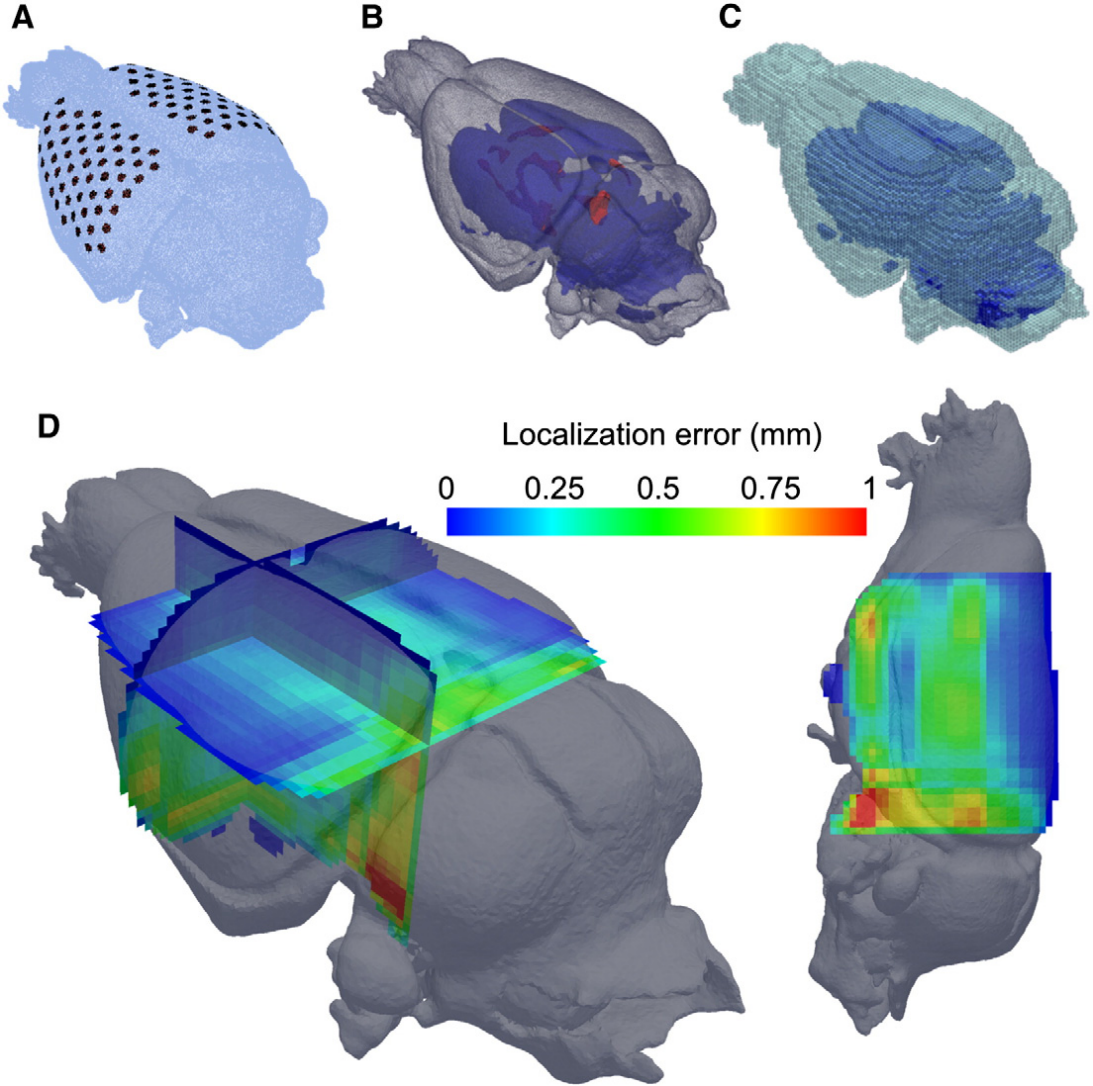
Accuracy of image reconstruction with a 120 contact electrode array, as determined in simulations. A) 15 M-elements forward mesh, B) comprising anatomically accurate material properties: gray matter (transparent gray), white matter (blue), and CSF (red), and C) a 100 k hexahedral elements inverse mesh, were used to access the accuracy. D) Localization error was calculated throughout the volume at 1000 locations in simulation. A superior-lateral (left) and left sagittal (right) view is shown of the mesh with rasterization planes through the mesh color-coded according to the localization error determined by difference between real and reconstructed perturbation location (1% conductivity change, 0.5 mm diameter). The transverse plane in the left subplot is positioned 3 mm below vertex of the brain. The resolution in most of the neocortex is < 250 μm, and is < 500 μm throughout most of the brain (with the exclusion of structures adjacent to the skull-base and ventricles).

With the epicortical arrays described above the injected current density is highest near the cortical surface, so that the volume of high accuracy is limited, especially with respect to depth. Computer simulations have indicated that within the total imaging volume of 70 mm^3^ the positional accuracy is < 0.6 mm, with high accuracy, < 0.2 mm, in a 28 mm^3^ volume (5 × 4 × 1.4 mm) under the array and a poorer resolution further from the center of the array (Aristovich et al., 2014): the same spatial resolution could be expected in the work presented here as the same methodology was employed in the *in vivo* studies. It would clearly be preferable to be able to image neural activity throughout the entire rat brain, as the simultaneous measurement of cortical and subcortical activity would broaden the hypotheses that might be explored with EIT. To address this future aim, simulations were undertaken to characterize the spatial accuracy of EIT imaging throughout the rat entire brain if two 60 electrode arrays were placed on the cortical hemispheres, one on each side; the methodology of the simulations was similar to that published previously (Aristovich et al., 2014), and is described in the Experimental procedures section. This indicated that the resolution in most of the neocortex is < 250 μm, and is < 500 μm throughout most of the brain, with the exception of structures adjacent to the skull-base and ventricles (Fig. 6). Such resolution would enable imaging of synchronous activation of subcortical structures, such as the hippocampus or larger thalamic nuclei (Paxinos and Watson, 2013). This would allow simultaneous imaging of subcortical and cortical activity, which could illuminate the underlying mechanisms of subcortical–cortical interactions and permit mathematical modeling and analysis of 3D circuit behavior.

### Discussion

Here we have presented the first ever tomographic images of neural activity reconstructed using EIT, acquired simultaneously over a large volume (70 mm^3^) using non-penetrating surface electrodes, and with a spatial and temporal resolution of c. 200 μm and ≤ 2 ms, respectively. The combination of such imaging properties has not been previously achieved with any neuroimaging technique; this illustrates the unique potential of EIT as a neuroimaging modality. The imaging was of activity following stimulation of two separate foci within the S1 whisker representation, with reproducibility shown within and across subjects for these different foci. Conductivity changes in the EIT images correlated significantly with CSDA. Unlike CSDA, such characteristics could be extracted from anywhere in the imaging volume.

As compared to the ‘ideal’ neuroimaging technique, EIT imaging of neural activity, as presented in this study, has multiple limitations. These include that the current *in vivo* findings were limited to S1, had a somewhat limited depth penetration and had a 2 ms and mesoscopic resolution. However, the simulations presented here demonstrate this methodology could be extended to imaging of the entirety of the brain with minor alterations and improvements in temporal resolution achieved with hardware and signal processing improvement.

### Validation: *in vivo* and comparison to literature

The use of EIT to image neural activity was validated by simultaneous *in vivo* LFP recordings and CSDA. This validation was primarily achieved by determining that features of the EIT images — onset time and peak amplitude — significantly correlated with the same features in CSDA, with both techniques matching previously published findings. Onset times significantly correlated and were comparable to previously published findings (Einevoll et al., 2007); disparity with the published literature was most likely attributable to dissimilar surgical and anesthetic preparation, in addition to physiological variations. The onset of activity was located at a similar depth from the cortical surface for both EIT and CSDA, at a depth approximating layer IV, which is a principal target of thalamocortical projections and site of initial activity. A second site of activity onset, in layer Vb, was identified with CSDA, which matches previously published findings (Armstrong-James et al., 1992, Petersen, 2007); however, this second site was not clearly distinguished in the EIT images. The similarities between EIT and CSDA, and between EIT and the rat neurophysiological literature suggest that this difference should not undermine the potential applicability of EIT. The cause of the differences is not entirely clear. It could have been because the sites are separated by 200–400 μm which is approaching the spatial resolution of EIT in depth, or because the mechanism of conductivity changes differs from that underlying LFP. A potential advantage of impedance measurement is that it can complement LFP recording. Conductivity change occurs as ion channels open; both excitatory activity and inhibitory activity result in a conductivity change. In contrast, summed positive and negative potential fields may cancel and not be measured with LFP recording. Apart from this discrepancy, the onset latency at other depths was consistent between EIT and CSDA: activity spread to infragranular and supragranular layers within approximately 1 and 3.5 ms, respectively. The same pattern of initial activity in layer IV spreading to infragranular and supragranular is well documented (Armstrong-James et al., 1992, Einevoll et al., 2007, Roy et al., 2011), although supragranular spread is typically found to precede spread to infragranular layers. However, in our study, simultaneous CSDA had the same chronology of spread as EIT.

The lateral spread of activity was explored with the generation of spatiotemporal trajectories. These indicated that lateral spread was predominantly in layers II/III and had a preference for row-wise propagation. In addition to this, layers II/III were found overall to have the greatest number of spatiotemporal connections. While these findings could not be validated *in vivo* in our paradigm they are in good agreement with the established literature (Feldmeyer et al., 2006, Holmgren et al., 2003, Lustig et al., 2013, Petersen, 2007, Petersen and Sakmann, 2001, Petersen et al., 2003).

### Generalization of the method

These findings indicate that EIT can be employed to examine translaminar activity, simultaneously at multiple cortical sites, with high spatial and temporal resolutions, and that the characteristics of this activity are significantly matched to that identified with CSDA, but can be collected over an unprecedented volume. While the current *in vivo* work was limited to S1, the simulations presented here demonstrate this methodology could be extended to imaging of the entirety of the brain with minor alterations. The result is a powerful, versatile, and minimally invasive tool for the imaging and investigation of neural activity.

The method described in this paper can be customized for specific experimental needs, including imaging of the entire cortex, by adjusting certain parameters and can be reproduced with off-the-shelf hardware. The customization parameters are: 1) size, number, or relative placement of electrodes, 2) the sequence and number of current injection sites, 3) number of repetitions, and 4) image reconstruction technique (Adler and Lionheart, 2006, Adler et al., 2009, Aristovich et al., 2014, Jehl et al., 2014, Lionheart, 2004). Alteration of these parameters allows for optimization of several imaging characteristics, including, but not limited to, spatial resolution, imaging volume and sensitivity, and recording duration.

The presented method can be also applied simultaneously with other neuroscientific tools such as inverse source reconstruction, depth electrode recordings and optical methods, especially with the use of recently available transparent electrodes (Kuzum et al., 2014). A multi-modal approach could offer cellular level resolution at predefined locations within the cortex while simultaneously providing imaging of rapid neural activity throughout the rest of the brain.

## Experimental procedures

### Animal preparation

Four female Sprague-Dawley adult rats weighing 300 to 450 g were used for recordings. Anesthesia was induced with a mixture of O_2_ and isoflurane. Carprofen was administered subcutaneously for pain relief, an endotracheal tube (Vet Tech Solutions Ltd., UK) was introduced following a tracheostomy and mechanical ventilation, using a Harvard Apparatus Inspira Ventilator (Harvard Apparatus, Ltd, UK), was commenced with a 50/50% gas mixture of O_2_ and air. Arterial and venous access was established through cannulation (BD Insyte/Vialon, Becton, Dickinson UK Ltd.) of the right femoral vessels. The arterial blood pressure was monitored (Cardiocap 5, Datex Ohmeda) and the mean arterial pressure (MAP) kept between 90 and 110 mm Hg using labetalol and adrenaline as necessary. Once intravenous access had been established, a constant infusion of propofol was initiated. Rats were then fixed in a stereotactic frame (Narishige International Ltd., UK), their head shaved and the scalp incised. The insertion of the temporal muscle(s) was cauterized using a bipolar cauterization system (Malis CMC 2, Codman, USA). A craniotomy was then performed using a bone drill (Ideal Micro-DrillTM) with frequent irrigation using 0.9% saline so that the paramedical edge extended 2 to 3 mm rostral to bregma and immediately rostral of lambda, and the lateral aspect was at the junction of the zygoma to temporal bone, so that the resulting craniotomy was trapezoidal in shape. The bone flap was then lifted and the dura incised with micro scissors and reflected. Then ISOI was performed to aid electrode placement, after which a 30-electrode array was then placed over PMBSF using a micromanipulator (SM-15 Micromanipulator, Narishige International Ltd., UK) and a linear, 16-contact electrode was inserted, through a hole in the middle of the EIT array. Finally, a 2.25 cm^2^ silver–silver chloride ground electrode was placed under the nuchal skin. Procedures were performed on a vibration isolated table (Thorlabs Inc., USA) and throughout experiments the core body temperature of the rat was controlled with a homeothermic heating unit (Harvard Apparatus, Kent, UK). All animal works undertaken in this study were approved by the UK Home Office and in accordance with its regulations (Project number: PPL 70/7450).

ISOI was performed using a previously published paradigm and data analysis (Berwick et al., 2008), and the following hardware was used: a 1.3 megapixel monochrome charge-coupled device camera (STC-MB133USB, Sentec, Sensor Technologies America, Inc., USA) with a macroscopic zoom lens (MLM3X-MP, Computar, CBC Corp., USA) and a high brightness, ripple-free LED light source (Schott KL 1500 LED, Schott North America, Inc., USA) with a green insert filter (peak 520 nm, full width at half maximum ± 30 nm).

### EIT hardware, data acquisition and protocols

Data were collected using a laser cut stainless-steel foil on silicone rubber 30 contact electrode array covering 5 × 7 mm. The electrode contacts were circular, 0.6 mm in diameter, arranged hexagonally at a 1.2 mm center-to-center distance. The connecting track width was 150 μm with an inter-track spacing of 35 μm. The stainless-steel foil was 12.5 μm and the total thickness of the array was 220 to 250 μm. The electrodes were platinized to reduce contact impedance and noise from the electrode–electrolyte interface.

Evoked responses were induced using a custom made mechanical whisker stimulator: a piezoelectric stimulator, based on an actuator (PL140.10, PI ceramic, Germany) was used to displace whiskers in the craniocaudal axis at 1 Hz. The displacement was 4 mm and the rise time was 5 ms. One of two groups of four whiskers were tied together and attached to the piezoelectric stimulator. The groups comprised whisker barrels δ, γ, E1, and D1, or D2, C2, D3 and C3, which were diagonally adjacent. In each rat both groups were stimulated twice, therefore yielding 4 images per rat, this was undertaken in 4 rats (*n* = 16, *N* = 4). Data were recorded in trials, each consisted of rapid forward whisker deflection, 0.5 s interstimulus interval, rapid backwards whisker deflection, and a second 0.5 s interstimulus interval, resulting in a 1 s trial; the stimulator also generated a trigger signal which was used to separate the trials. All statistical analyses used data from both deflections, the only exception was spatiotemporal trajectories for which forward deflection data was used.

Current was injected using a custom built current source ‘UCL-CS2’ (Oh et al., 2011) through a single pair of electrodes at a time and the pair was switched via a multiplexer with the remaining electrodes recording the resultant voltage using a BioSemi amplification and acquisition system (ActiveTwo, BioSemi, Netherlands) with a sampling rate of 8 kHz at 24 bits. A heuristic current injection protocol based on the best depth distinguishability was employed (Kao et al., 2003). Each current injection contained 15 consecutive 1 s trials therefore totaling 900 s. The injected current was 50 μA in amplitude at a frequency of 1725 Hz, and the impedance changes were demodulated with a ± 500 Hz bandwidth around this carrier frequency, giving 2 ms time resolution.

### Controls

**Supplementary Fig. 1:**
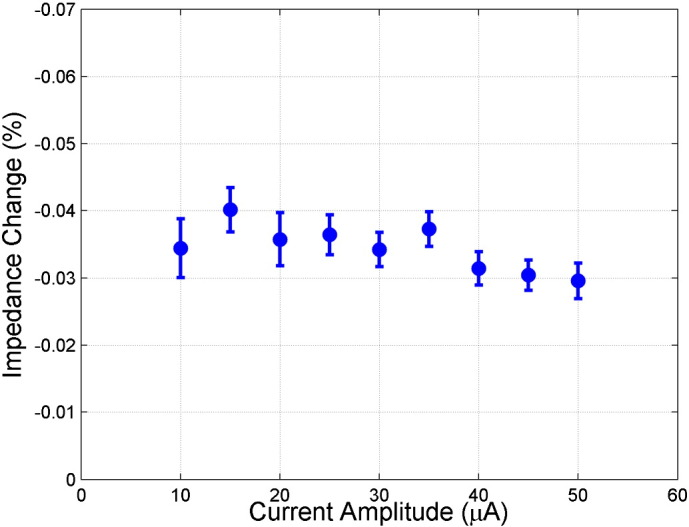
Variable current amplitude control. The mean (± 1 SE) over all repeats (*n* = 24) in 2 rats. There was no significant difference between the percentage impedance changes across current amplitudes (*p* > 0.3 using one-way ANOVA).

Control recordings were made in order to ensure the validity and reproducibility of data: 1) no stimulation controls were collected in 2 rats where data acquisition was performed with the whisker stimulator being disconnected from the whiskers. 2) Dead rat controls were undertaken in 2 dead rats. 3) Current amplitude controls were performed in 2 rats where the injected current amplitude was varied from 10 to 70 μA, and single channel impedance measurements were made. 4) Current phase relation was excluded by randomizing the phase with respect to the beginning of each stimulus. No significant impedance change occurred when no stimulation was provided, nor in dead rats (*p* > 0.05, one-side unpaired *t*-test), and variation of the injected current amplitude revealed that the percentage impedance change did not significantly alter with amplitude (*p* > 0.05, one-way ANOVA, Supplementary Fig. 1).

### Data processing and image reconstruction

Measured boundary voltages were processed to reject noisy channels. Channels with boundary voltages < 100 μV were rejected, in addition channels were rejected based upon their interstimulus standard deviation (SD) after averaging. SD thresholding was set so that 90% of the channels with an impedance change three times greater than the average interstimulus SD were preserved.

Images were reconstructed with the real component of voltage differences using a time difference approach, where the baseline boundary voltage was taken as the mean interstimulus boundary voltage. The image reconstruction has been described previously (Aristovich et al., 2014, Jehl et al., 2014), but broadly entailed reconstruction using a finite element method approach with scaled MRI-based atlas head models and zeroth-order Tikhonov regularization with a noise-based correction. Photogrammetry was used to measure the position of the electrode array to ensure accurate image reconstruction (Russell et al., 2005).

### Local field potentials (LFP) and current source density analysis (CSDA)

Recordings were made with a single shank linear silicone probe. Comprising 16 iridium contacts, 30 μm in diameter spaced 100 μm apart (A16, NeuroNexus Technologies, Ann Arbor, MI, USA). The probe was positioned orthogonal to the exposed cortical surface using a micromanipulator and advanced until, under microscopic assessment, the uppermost electrode entered the pial tissue. Confirmatory recordings were then made to ensure correct electrode placement. LFP data were passed through a headstage amplifier (E2a, Plexon, TX, USA), with unity gain, and recorded with a BioSemi EEG system (ActiveTwo, BioSemi, Netherlands) with a sampling rate of 8 kHz. LFPs and EIT data were collected simultaneously.

Data were low pass filtered at 500 Hz and averaged. The data were spatially arranged over depth and linearly interpolated onto 50 μm segments, resulting in a total of 32 segments. Spatial smoothing was then performed with a Gaussian kernel with a full width at half maximum of 100 μm and CSDA undertaken (Mitzdorf, 1985, Nicholson and Freeman, 1975). To remove oscillation in the resultant CSDA and prevent incorrect analysis, only significant sources and sinks were considered. Significance was determined as data being more than three standard deviations of the data from the interstimulus period. For each depth, and each whisker deflection direction, the peak response was detected and the onset of the CSDA was defined as the first time point before this peak at which the changes became significant (> 3 SD of the interstimulus period). In addition, the overall earliest onset and its depth were computed.

### Image analysis and cross-validation

#### Translaminar propagation

Conductivity changes over time and depth in a region of interest (ROI) at the LFP electrode location was compared to CSDA. The ROI comprised a vertical column, 200 × 200 × 2000 μm, divided into 50 μm thick layers and the onset and peak conductivity change per layer were determined. The peak response was detected and the onset of the conductivity change defined as the first time point before this at which changes became significant (i.e. > 3 interstimulus SD). These depth-wise onset times were then normalized to the earliest onset of activity. For EIT and CSDA separately the onset time for each depth was tested, using a two-sided *t*-test (*p* < 0.05), for whether it was significantly different from the earliest onset time. Additionally, each depth segments onset time was tested, using a paired *t*-test (*p* < 0.05), to determine if a given depth had a significantly different onset time between EIT and CSDA. Both *t*-tests were corrected using a Bonferroni correction. The depth of activity for this and subsequent analysis were compared to published laminar thicknesses in order to infer the lamina from which activity arose or spread into (Armstrong-James et al., 1992, Di et al., 1990, Krieg, 1946, Simons, 1978, Zilles, 1985). In addition to this, the peak amplitude of the EIT and CSDA over all depths and time points was detected.

#### 4D spatiotemporal trajectories

Spatiotemporal trajectories were computed in order to examine the 4D propagation of activity. First, the center of activity was identified for each image. Then the following set of equations was solved in order to compute trajectories:

$$v=\nabla \left(\frac{d\sigma \left(r,t\right)}{dt}\right)\text{;}$$

(1) $$\frac{dr}{dt}=v\text{,}$$

where r = r(*t*) is the trajectory, *σ* is the conductivity change, and v = v(r, *t*) is the gradient of rate of change of *σ* (similar to the velocity field in mechanics). Applying a Euler 1st order computational scheme for a single trajectory, it can be computed via the following set of iterative equations:

$$r_{0}=r_{\mathrm{start}}\text{;}$$

$$r_{i}=r_{i-1}+\nabla \left(\frac{d\sigma \left(r_{i-1},t_{i-1}\right)}{dt}\right)\left(t_{i}-t_{i-1}\right)\text{;}$$

(2) $$i=\bar{1:N}\text{,}$$

where *i* is a computational step in the temporal domain, and *δt* = *t*_*i*_ − *t*_*i* − 1_ is the integration step, which can be estimated for each image using the longest step condition:

(3) $$\delta t=\frac{r_{z}}{\arg\max_{z,\;t}\left(\nabla \frac{d\sigma }{dt}\right)}\text{,}$$

where *r*_*z*_ = 50 μm is the longest step for a given sampling frequency, determined by the average velocity of translaminar propagation. Eq. (2) is solved for 1000 trajectories with the start points equally distributed from a ROI 500 × 500 × 500 μm around the center of activity and displayed in 3D as connected curves.

### Entire brain volume spatial resolution simulations

Simulations were undertaken to characterize the spatial accuracy of EIT imaging throughout the rat entire brain if two 60 electrode arrays are placed on the cortex. Simulations were performed in an anatomically realistic rat brain model obtained from an MRI image, with electrode arrays placed bilaterally on the cortex. The background conductivity was constant and isotropic throughout the gray matter (0.3 S m^− 1^), white matter (0.15 S m^− 1^), and CSF-ventricles (1.75 S m^− 1^). The modeled conductivity change was a sphere 0.5 mm in diameter, approximating larger thalamic nuclei (Paxinos and Watson, 2013), with conductivity 1% higher than the background conductivity (0.303 S m^− 1^), typical of the reconstructed conductivity change. It was placed in 1000 locations throughout the cortex, excluding the olfactory bulbs and cerebellum, in steps of 0.5 mm along each axis. Realistic additive Gaussian noise with zero mean and a standard deviation of 0.5 μV was added to all simulated voltage changes. Each perturbation was reconstructed using the methods already detailed and the distance between the modeled and reconstructed perturbation center of mass expressed as an error and projected onto the rat brain model over the modeled location.

The following are the supplementary data related to this article.

**Supplementary Fig. 2:**
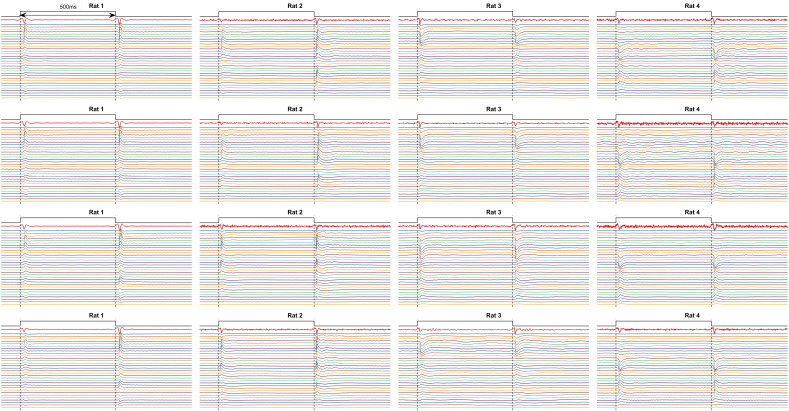
Summary of EIT and SCDA raw data of 16 recordings. Each subplot represents single recording with stimulation signal (black), average raw EIT signal before image reconstruction in single channel (red), and CSDA data over depth for 32 interpolation points (pale colors). The signals are normalized between recordings to allow visual comparison.

Supplementary Video 1 Example of EIT image during forward deflection of whisker group 1 (δ, γ, E1, and D1). The video shows onset of activity at 7 ms occurring at c. 800 μm beneath the pial surface, and over the ensuing milliseconds encompassing a larger volume reaching a maximum at 10–11 ms, following which the activity spreads to adjacent areas in S1 and disappears at 17 ms. The color code is identical to Fig. 2.

Supplementary Video 2 An example video of a 4D spatiotemporal trajectory, calculated from data collected using a planar electrode array and following mechanical whisker stimulation. Color indicates time on the scale from 7 to 15 ms (see Fig. 5).

## Author contributions

K.Y.A and B.C.P. designed and conducted the research, analyzed the data, and wrote the paper; H.K. provided instrumentation; G.S.D.S. designed some components of the reconstruction routine; A.McE. and D.S.H supervised the work and advised on the paper.

## Figures and Tables

**Table 1:** Comparison of parameters (mean ± 1SE) extracted from a region of interest in EIT images (centered on the LFP electrode location) and from the CSDA.

	EIT	CSDA
Onset time	8.1 ± 0.4 ms	8.7 ± 0.5 ms
Onset depth	790 ± 70 μm	730 ± 50 μm
Amplitude	0.043 ± 0.01%	4.3 ± 0.7 mV mm^2^

## References

[bb0010] Adler A., Lionheart W.R.B. (2006). Uses and abuses of EIDORS: an extensible software base for EIT. Physiol. Meas..

[bb0005] Adler A., Arnold J.H., Bayford R., Borsic A., Brown B., Dixon P., Faes T.J.C., Frerichs I., Gagnon H., Gärber Y., Grychtol B., Hahn G., Lionheart W.R.B., Malik A., Patterson R.P., Stocks J., Tizzard A., Weiler N., Wolf G.K. (2009). GREIT: a unified approach to 2D linear EIT reconstruction of lung images. Physiol. Meas..

[bb0015] Ahrens M.B., Orger M.B., Robson D.N., Li J.M., Keller P.J. (2013). Whole-brain functional imaging at cellular resolution using light-sheet microscopy. Nat. Methods.

[bb0020] Aristovich K.Y., Santos G.S. Dos, Packham B.C., Holder D.S. (2014). A method for reconstructing tomographic images of evoked neural activity with electrical impedance tomography using intracranial planar arrays. Physiol. Meas..

[bb0025] Armstrong-James M., Callahan C.A., Friedman M.A. (1991). Thalamo-cortical processing of vibrissal information in the rat. I. Intracortical origins of surround but not centre-receptive fields of layer IV neurones in the rat S1 barrel. J. Comp. Neurol..

[bb0030] Armstrong-James M., Fox K., Das-Gupta A. (1992). Flow of excitation within rat barrel cortex on striking a single vibrissa. J. Neurophysiol..

[bb0035] Bayford R.H. (2006). Bioimpedance tomography (electrical impedance tomography). Annu. Rev. Biomed. Eng..

[bb0040] Berwick J., Johnston D., Jones M., Martindale J., Martin C., Kennerley A.J., Redgrave P., Mayhew J.E.W. (2008). Fine detail of neurovascular coupling revealed by spatiotemporal analysis of the hemodynamic response to single whisker stimulation in rat barrel cortex. J. Neurophysiol..

[bb0045] Borsic A., Halter R., Wan Y., Hartov A., Paulsen K.D. (2010). Electrical impedance tomography reconstruction for three-dimensional imaging of the prostate. Physiol. Meas..

[bb0050] Cole K.S., Curtis H.J. (1939). Electric impedance of the squid giant axon during activity. J. Gen. Physiol..

[bb0055] Di S., Baumgartner C., Barth D.S. (1990). Laminar analysis of extracellular in rat vibrissa/barrel cortex field potentials. J. Neurophysiol..

[bb0060] Diamond M.E., von Heimendahl M., Knutsen P.M., Kleinfeld D., Ahissar E. (2008). “Where” and “what” in the whisker sensorimotor system. Nat. Rev. Neurosci..

[bb0065] Einevoll G.T., Pettersen K.H., Devor A., Ulbert I., Halgren E., Dale A.M. (2007). Laminar population analysis: estimating firing rates and evoked synaptic activity from multielectrode recordings in rat barrel cortex. J. Neurophysiol..

[bb0070] Feldmeyer D., Lübke J., Sakmann B. (2006). Efficacy and connectivity of intracolumnar pairs of layer 2/3 pyramidal cells in the barrel cortex of juvenile rats. J. Physiol..

[bb0075] Frerichs I. (2000). Electrical impedance tomography (EIT) in applications related to lung and ventilation: a review of experimental and clinical activities. Physiol. Meas..

[bb0080] Freygang W.H., Landau W.M. (1955). Some relations between resistivity and electrical activity in the cerebral cortex of the cat. J. Cell. Comp. Physiol..

[bb0085] Heeger D.J., Ress D. (2002). What does fMRI tell us about neuronal activity?. Nat. Rev. Neurosci..

[bb0090] Henderson R.P., Webster J.G. (1978). An impedance camera for spatially specific measurements of the thorax. IEEE Trans. Biomed. Eng..

[bb0095] Hillman E.M.C. (2007). Optical brain imaging *in vivo*: techniques and applications from animal to man. J. Biomed. Opt..

[bb0100] Holmgren C., Harkany T., Svennenfors B., Zilberter Y. (2003). Pyramidal cell communication within local networks in layer 2/3 of rat neocortex. J. Physiol..

[bb0105] Jehl M., Dedner A., Betcke T., Aristovich K., Klofkorn R., Holder D. (2014). A fast parallel solver for the forward problem in electrical impedance tomography. IEEE Trans. Biomed. Eng. PP.

[bb0110] Kajikawa Y., Schroeder C.E. (2011). How local is the local field potential?. Neuron.

[bb0115] Kao T.-J., Newell J.C., Saulnier G.J., Isaacson D. (2003). Distinguishability of inhomogeneities using planar electrode arrays and different patterns of applied excitation. Physiol. Meas..

[bb0120] Klivington K.A., Galambos R. (1967). Resistance shifts accompanying the evoked cortical response in the cat. Science.

[bb0125] Kopell N.J., Gritton H.J., Whittington M.A., Kramer M.A. (2014). Beyond the connectome: the dynome. Neuron.

[bb0130] Krieg W.J.S. (1946). Connections of the cerebral cortex: I The albino rat. B Structure of the cortical atlas. J. Comp. Neuro.

[bb0135] Kuzum D., Takano H., Shim E., Reed J.C., Juul H., Richardson A.G., de Vries J., Bink H., Dichter M.A., Lucas T.H., Coulter D.A., Cubukcu E., Litt B. (2014). Transparent and flexible low noise graphene electrodes for simultaneous electrophysiology and neuroimaging. Nat. Commun..

[bb0140] Lionheart W.R.B. (2004). EIT reconstruction algorithms: pitfalls, challenges and recent developments. Physiol. Meas..

[bb0145] Liston A., Bayford R., Holder D. (2012). A cable theory based biophysical model of resistance change in crab peripheral nerve and human cerebral cortex during neuronal depolarisation: implications for electrical impedance tomography of fast neural activity in the brain. Med. Biol. Eng. Comput..

[bb0150] Luecke T., Corradi F., Pelosi P. (2012). Lung imaging for titration of mechanical ventilation. Curr. Opin. Anaesthesiol..

[bb0155] Lustig B.R., Friedman R.M., Winberry J.E., Ebner F.F., Roe A.W. (2013). Voltage-sensitive dye imaging reveals shifting spatiotemporal spread of whisker-induced activity in rat barrel cortex. J. Neurophysiol..

[bb0160] Malone E., Jehl M., Arridge S., Betcke T., Holder D. (2014). Stroke type differentiation using spectrally constrained multifrequency EIT: evaluation of feasibility in a realistic head model. Physiol. Meas..

[bb0165] Maynard E.M., Nordhausen C.T., Normann R.A. (1997). The Utah intracortical electrode array: a recording structure for potential brain-computer interfaces. Electroencephalogr. Clin. Neurophysiol..

[bb0170] Metherall P., Barber D.C., Smallwood R.H., Brown B.H. (1996). Three-dimensional electrical impedance tomography. Nature.

[bb0175] Mitzdorf U. (1985). Current source-density method and application in cat cerebral cortex: investigation of evoked potentials and EEG phenomena. Physiol. Rev..

[bb0180] Nguyen D.T., Jin C., Thiagalingam A., McEwan A.L. (2012). A review on electrical impedance tomography for pulmonary perfusion imaging. Physiol. Meas..

[bb0185] Nicholson C., Freeman J.A. (1975). Theory of current source-density analysis and determination of conductivity tensor for anuran cerebellum. J. Neurophysiol..

[bb0190] Oh T., Gilad O., Ghosh A., Schuettler M., Holder D.S. (2011). A novel method for recording neuronal depolarization with recording at 125–825 Hz: implications for imaging fast neural activity in the brain with electrical impedance tomography. Med. Biol. Eng. Comput..

[bb0195] Paxinos G., Watson C. (2013). The rat brain in stereotaxic coordinates. The Rat Brain in Stereotaxic Coordinates.

[bb0200] Petersen C.C.H. (2007). The functional organization of the barrel cortex. Neuron.

[bb0210] Petersen C.C.H., Sakmann B. (2001). Functionally independent columns of rat somatosensory barrel cortex revealed with voltage-sensitive dye imaging. J. Neurosci..

[bb0205] Petersen C.C.H., Grinvald A., Sakmann B. (2003). Spatiotemporal dynamics of sensory responses in layer 2/3 of rat barrel cortex measured *in vivo* by voltage-sensitive dye imaging combined with whole-cell voltage recordings and neuron reconstructions. J. Neurosci..

[bb0215] Roy N.C., Bessaih T., Contreras D. (2011). Comprehensive mapping of whisker-evoked responses reveals broad, sharply tuned thalamocortical input to layer 4 of barrel cortex. J. Neurophysiol..

[bb0220] Russell G.S., Jeffrey Eriksen K., Poolman P., Luu P., Tucker D.M. (2005). Geodesic photogrammetry for localizing sensor positions in dense-array EEG. Clin. Neurophysiol..

[bb0225] Simons D.J. (1978). Response properties of vibrissa units in rat SI somatosensory neocortex. J. Neurophysiol..

[bb0230] Smallwood R.H., Mangnall Y.F., Leathard A.D. (1994). Transport of gastric contents (electric impedance imaging). Physiol. Meas..

[bb0235] Smith T.G., Wuerker R.B., Frank K. (1967). Membrane impedance changes during synaptic transmission in cat spinal motoneurons. J. Neurophysiol..

[bb0240] Somersalo E., Cheney M., Isaacson D. (1992). Existence and uniqueness for electrode models for electric current computed tomography. SIAM J. Appl. Math..

[bb0245] Sporns O. (2014). Contributions and challenges for network models in cognitive neuroscience. Nat. Neurosci..

[bb0250] Tsukahara N., Fuller D.R. (1969). Conductance changes during pyramidally induced postsynaptic potentials in red nucleus neurons. J. Neurophysiol..

[bb0255] Wilent W.B., Contreras D. (2005). Dynamics of excitation and inhibition underlying stimulus selectivity in rat somatosensory cortex. Nat. Neurosci..

[bb0260] Zilles K. (1985). The Cortex of the Rat: A Stereotaxic Atlas.

[bb0265] Zou Y., Guo Z. (2003). A review of electrical impedance techniques for breast cancer detection. Med. Eng. Phys..

