# Supplementary figures and images for: Imaging fast electrical activity in the brain with electrical impedance tomography
Source: Neuroimage. 2016 Jan 1;124(Pt A):204–13. doi: 10.1016/j.neuroimage.2015.08.071 (PMC4655915; doi:10.1016/j.neuroimage.2015.08.071)

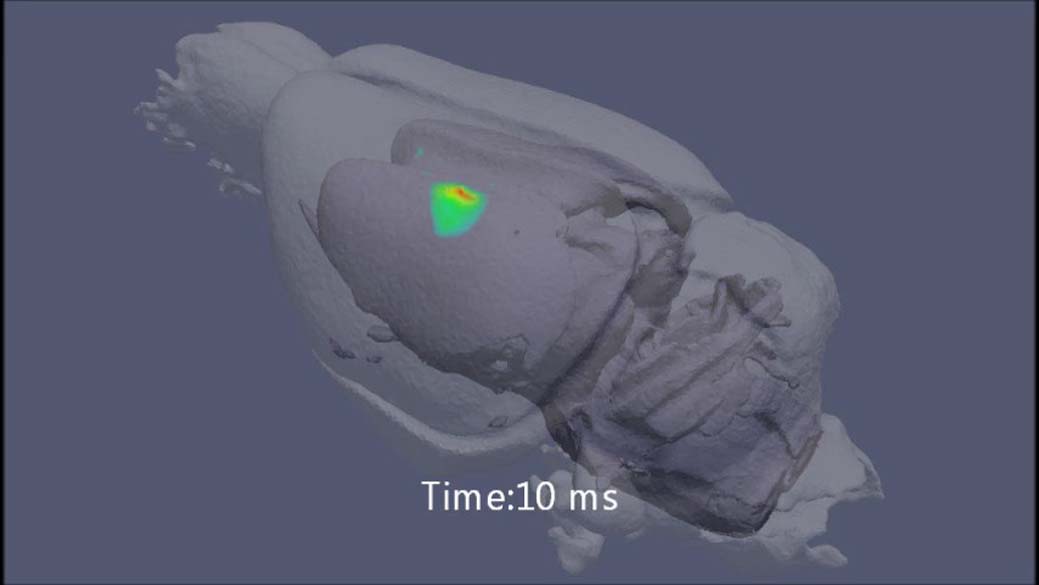

Supplement: Supplementary Video 1 — Example of EIT image during forward deflection of whisker group 1 (δ, γ, E1, and D1). The video shows onset of activity at 7 ms occurring at c. 800 μm beneath the pial surface, and over the ensuing milliseconds encompassing a larger volume reaching a maximum at 10–11 ms, following which the activity spreads to adjacent areas in S1 and disappears at 17 ms. The color code is identical to Fig. 2. [file mmc1.jpg]

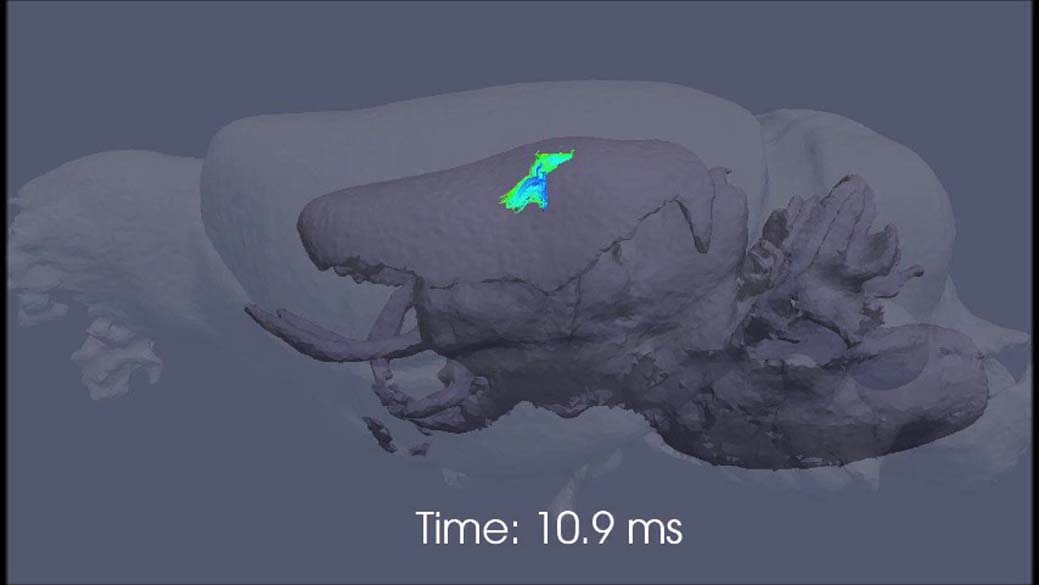

Supplement: Supplementary Video 2 — An example video of a 4D spatiotemporal trajectory, calculated from data collected using a planar electrode array and following mechanical whisker stimulation. Color indicates time on the scale from 7 to 15 ms (see Fig. 5). [file mmc2.jpg]
